# Post-diagnostic support for persons with young-onset dementia – a retrospective analysis based on data from the Swedish dementia registry SveDem

**DOI:** 10.1186/s12913-024-11108-7

**Published:** 2024-05-21

**Authors:** Fanny Kårelind, Deborah Finkel, Steven H Zarit, Helle Wijk, Therese Bielsten, Linda Johansson

**Affiliations:** 1 Studies on Integrated Health and Welfare (SIHW), Institute of Gerontology, School of Health and Welfare, Jönköping University, Jönköping, Sweden; 2 Institute of Gerontology, School of Health and Welfare, Jönköping University, Jönköping, Sweden; 3 Institute of Health and Care Science, The Sahlgrenska Academy at Gothenburg University, Gothenburg, Sweden; 4 Center for Economic and Social Research, University of Southern California, Los Angeles, USA; 5 Department of Human Development and Family Studies, Penn State University, University Park, USA; 6 Department of Architecture and Civil Engineering, Chalmers University of Technology, Gothenburg, Sweden

**Keywords:** Dementia care, Formal support services, Memory clinics, Quality registry, Young-onset dementia, YOD, Early-onset dementia

## Abstract

**Background:**

Approximately 3.9 million persons worldwide have young-onset dementia. Symptoms related to young-onset dementia present distinct challenges related to finances, employment, and family. To provide tailored support, it is important to gain knowledge about the formal support available for persons with young-onset dementia. Therefore, this paper aims to describe formal support for persons with young-onset dementia in Sweden and the factors influencing this support.

**Methods:**

This retrospective study used data on persons under 65 years of age (*n* = 284) from The Swedish Registry for Cognitive/Dementia Disorders (SveDem) between 2021 and 2022. SveDem was established to monitor the quality of dementia care in Sweden. Characteristics of participants were obtained, including age, sex, dementia diagnosis, MMSE, medications, accommodation, and care setting. Descriptive statistics and logistic regression were used to test for associations between participant characteristics and post-diagnostic support.

**Results:**

Information and educational support were usually offered to the person with young-onset dementia (90.1%) and their family (78.9%). Approximately half of the sample were offered contact with a dementia nurse (49.3%), counsellor (51.4%), or needs assessor (47.9%). A minority (28.5%) were offered cognitive aids. Six regression models were conducted based on participant characteristics to predict the likelihood that persons were offered support. Support was not predicted by age, sex, children at home, accommodation, or medications. Lower MMSE scores (*p* < .05) and home help (*p* < .05) were significantly associated with offer of a needs assessor. Living together was a significant predictor (*p* < .01) for information and educational support offered to the family. Care setting significantly predicted (*p* < .01) an offer of information and educational support for the person and family members, as well as contact with a counsellor.

**Conclusion:**

This study indicates potential formal support shortages for persons with young-onset dementia in some areas of dementia care. Despite equal support across most characteristics, disparities based on care setting highlight the importance of specialised dementia care. Pre-diagnostic support is minimal, indicating challenges for persons with young-onset dementia to access these services before diagnosis. While our study has identified areas in need of improvement, we recommend further research to understand the changing support needs of those with young-onset dementia.

## Background

### Diagnosis and prevalence overview

Dementia, affecting over 50 million globally, is a leading cause of disability in older persons [1]. It impacts cognitive function and can manifest in memory loss, disorientation, and communication challenges [2]. While age increases its risk, dementia can also affect younger persons [1]. Alzheimer’s disease is the most prevalent form of dementia, regardless of age for onset [3–5]. Still, persons with young-onset dementia are more likely than older adults to experience other types of dementia, such as frontotemporal and vascular dementia. Secondary causes for dementia are also more common for persons with young-onset dementia, such as alcohol-related dementia and traumatic injuries [6, 7]. The age cutoff for young-onset dementia is not based on biological ageing but on social markers of age [2], such as retirement age. Age 65 is also a standard cutoff for ageing research. The prevalence of young-onset dementia in Sweden is not precisely known, but estimates suggest 9,500 − 12,000 persons [8, 9]. The prevalence has steadily increased over time, likely due to increases in population size and improved diagnostic screening [10].

Receiving a dementia diagnosis at a younger age is often a shocking event that triggers many emotions in the person [11, 12], including anger, hopelessness, and grief. However, it can also bring a sense of relief by explaining the experienced symptoms [13–15]. Persons with dementia face challenges such as maintaining social relationships, managing daily activities [16], and increasing reliance on formal support [17]. Persons with young-onset dementia may face additional challenges, such as lost or reduced employment, leading to financial strain [18, 19]. Family members may also need to give up work to care for their relatives [4, 11, 18]. Persons with young-onset dementia are also more likely to have their children or adolescents still living in the home than persons with late-onset dementia [19]. In addition to the general stigma of living with dementia, research suggests that persons with young-onset dementia may experience stigma associated with their symptoms and diagnosis, which could lead to isolation [18]. Difficulty dealing with complex work responsibilities [20] can also increase the risk of stigma. These circumstances call for tailored care and support for persons with young-onset dementia, addressing their distinct challenges.

### Needs and support

While a cure for dementia remains elusive, research emphasises the positive impact of interventions on symptom management and the overall well-being of both persons with dementia and their families [21]. The challenges faced by persons with young-onset dementia underscore the need for tailored support services such as practical advice and information, support with daily activities, mental health support, and support for their families [18, 22, 23]. Psychosocial support is particularly important for addressing feelings of grief and loss of identity for persons with young-onset dementia [24]. Despite these needs, tailored support services are often lacking [18, 25, 26] leading persons with young-onset dementia feeling unsupported and neglected by service systems [22].

### Research context

In Sweden, dementia care is divided between two formal care systems: regions and municipalities. Regions provide healthcare in hospitals, primary care, and specialised outpatient services, like memory clinics, while municipalities provide home help, nursing homes, and home health care. There are two main laws governing the regions and the municipalities: The Health and Medical Care Act [HSL] [27] and the Social Services Act [SoL] [28]. HSL aims to promote equal healthcare, emphasising priority for those in most need. SoL promotes economic and social security, equal living conditions, and community participation. Another law impacting care for persons with young-onset dementia is The Act on Support and Service to Certain Functional Impairments [LSS] [29]. This law regulates services like personal assistance and residential care for those under 66 with significant functional impairment, ensuring equal living conditions and full participation in the community. Needs assessors (authorised social workers) determine access to support services within the SoL and LSS. Additionally, The Swedish National Guidelines for Care and Support for Dementia [30] set standards for diagnosis, treatment, and daily living recommendations, emphasising equality in care provision. Although several laws and regulations are in place for persons with dementia, there are indications that formal support lacks coordination. While research on coordinated support for persons with young-onset dementia in Sweden is limited, international studies suggest that support is poorly coordinated and communicated among providers [4, 31, 32].

Various services support the quality of life for persons with dementia, including memory clinics for diagnosis and treatment, daycare centres for socialisation, home help services, support groups, educational programs, and respite care. In Sweden, formal care and support for persons with dementia are mapped through the Swedish Registry of Cognitive/Dementia Disorders (SveDem), aligning with national quality guidelines for dementia [30]. Established in 2008, SveDem has registered 107,099 persons. It aims to enhance care quality across primary care, nursing homes, home health care, and specialist units, monitoring and evaluating care for persons with dementia. With extensive national coverage, it includes registrations from all memory clinics and 78% of primary care units in Sweden [33].

While research on young-onset dementia and age-specific support is increasing, there remains a significant gap in understanding the formal care and specific support types available to persons with young-onset dementia and their families. Additionally, there is a notable lack of knowledge regarding equality in care and support, essential for evaluating and ensuring appropriately tailored support and equal access to support services. Understanding the extent of support offered is essential for comprehending support needs. Therefore, investigating the current landscape of support services is critical to identifying potential gaps. To our knowledge, no other studies in Sweden describe this aspect, underlining the importance of shedding light on this issue. Therefore, this paper aims to use data from SveDem to describe post-diagnostic support offered to persons with young-onset dementia and the factors influencing this support.

## Methods

### Study design

This retrospective study analyses baseline data for all persons under 65 registered in The Swedish Registry for Cognitive/Dementia Disorders [SveDem] from April 2021 to May 2022. During this period, 284 new baseline registrations for this age group were entered in SveDem, with 261 from memory clinics and 23 from primary care units. The time frame chosen for this study was based on updates in the registry, which added new relevant variables in April 2021. Baseline data in SveDem are entered on newly diagnosed persons in the web-based registry by a local user, typically nurses or physicians, using the patient’s medical records as the source. Anything not entered in the medical records is considered “not performed” in SveDem [34]. Data included demographics, medication records, cognitive functioning, housing status, and support interventions.

### SveDem variables

Several participant characteristics were included to describe the sample and examine if any of these characteristics, including age, sex, living situation, level of functioning and number of medications (as a proxy for general health), would influence the different types of support offered. Two types of support variables are registered in SveDem – one is support already obtained at the time of diagnosis - *daycare*, *LSS* and *home help service.* The other is post-diagnostic support offered by the registration unit, which includes *information and educational support* (to a person or family member), contact with a *needs assessor, counsellor and dementia nurse*, and *cognitive aids*. Support offered to persons with young-onset dementia and their families is registered in SveDem. However, the database does not indicate whether the person used the support or if they were offered other support services not covered by the SveDem registry. Table 1 describes the variables included in the SveDem registry.

**Table 1:** Variables from SveDem

Variable	Description	Options
Accommodation*	Specifies whether the person resides in a nursing home or ordinary housing and the type of nursing home, if applicable.	Ordinary housing Nursing home, temporary Nursing home, permanent – not adapted for persons with dementia Nursing home, permanent- adapted for persons with dementia
Age	Person’s age at diagnosis.	Age in years
Care Setting	Specifies the care setting where registrations are made.	Memory Clinic Primary care centre
Children at home	Specifies if there are any children under 18 residing in the household.	Yes No
Cognitive Aids	Offered memory aids, including cognitive tools designed to assist persons in remembering dates, days of the week and medication schedules.	Yes No
Contact Counsellor	Offered contact with a counsellor to the person with dementia for emotional support and practical advice.	Yes No
Contact Dementia Nurse*	Offered contact with a dementia nurse to help and guide the person with dementia within health and social care.	Yes No No – no dementia nurse available
Contact Needs Assessor	Offered contact to a need assessor for support and care assessment.	Yes No
Dementia Diagnosis	Diagnosis received from a physician using the ICD classification system.	Mixed dementia Dementia UNS Alzheimer’s disease Dementia in Parkinson’s disease Frontotemporal dementia Lewy body dementia Mild cognitive disorder Alcohol dementia Other dementia
Daycare*	Daycare with activities for the person to interact socially with others.	Yes, adapted for persons with dementia Yes, adapted for persons with young-onset dementia Yes, but not adapted for persons with dementia No Do not know
Home Help Service	Home help services, including cooking, cleaning, hygiene, and meal assistance.	Yes No Do not know
(Information and Educational) Support to the person	Offered support to the person with HSL, including supportive conversations, support groups or educational resources.	Yes No
(Information and Educational) Support to family members	Offered support to family members, including conversations, education, or written information.	Yes No No – no relative available
Living arrangement	Indicates if the person is living alone or with another adult.	With another adult Living alone
Medications	Number of prescribed medications.	Number of medications
MMSE	Mini-Mental State Examination from diagnostic workup.	Score (0–30) Yes Not performed Not testable
Sex	Person’s sex	Male Female
Support According to LSS	Services, including personal assistance, daily activities, and residential care, provided to persons under 66 with extensive and permanent functional impairment.	Yes No Do not know

* Variable was dichotomised for analysis, see text for explanation.

### Data analysis

Descriptive statistics were used to provide a comprehensive overview of the data, emphasising its key characteristics. These included measures of central tendency, such as mean and median, to illustrate average values and measures of variability, such as standard deviation and range.

Logistic regression in SPSS version 28 was used to analyse the data and investigate the association between the predictor and outcome variables. Each model used one post-diagnostic support variable as the outcome and the characteristics of the persons with young-onset dementia as predictors. Collinear variables were removed from three models to enhance coefficient estimate reliability. Variables with more than two options were dichotomised for regression analysis. For instance, accommodation was recoded as ordinary housing or nursing home, with both nursing home options classified as “ Yes”. Similarly, daycare and dementia nurse variables were recoded as “Yes” or “No”. “No nurse available” was recorded as missing. Information and educational support for family members was coded as “Yes”, “No”, with “No Relative Available” recoded as missing.

The chi-square statistic was used to determine if the regression model explained a significant portion of the variance in the outcome variable. The significance level was set at 0.05, and the adjusted R^2^ value indicated the amount of variance explained.

## Results

### Demographic data

Table 2 presents the study population characteristics. About half of the sample were women (50.7%), with the majority (54.9%) falling in the 60–64-year age range, followed by the 55–59-year age range (29.6%). The overall mean age for the sample was 59.21 years, with a median of 60 years (SD 4.38). Most participants lived with another adult (70%), with only a small number residing in nursing homes (5.7%). A subset of the study population had children under 18 years living in their homes (9%), though the majority did not.

Alzheimer’s disease was the most prevalent diagnosis (52.5%), followed by frontotemporal dementia (11.6%) and mild cognitive disorder (10.9%). The mean number of prescribed medications was 4.11 (SD 3.35), ranging between 0 and 21 medications. The mean MMSE score was 22.61 (SD 4.59), ranging from 8 to 30. Finally, most persons (91.9%) were registered by memory clinics rather than primary care centres.

**Table 2:** Characteristics of persons with young-onset dementia (*n* = 284)

	Response categories (code)	*N* (*N* missing)	Valid per cent (%)
Age at diagnosis (years)			
	30–34	1	0.4
	40–44	1	0.4
	45–49	4	1.4
	50–54	38	13.4
	55–59	84	29.6
	60–64	156	54.9
Sex			
	Female (1)	144	50.7
	Male (2)	140	49.3
Dementia diagnosis			
	Mixed dementia	16	5.6
	Dementia UNS	15	5.3
	Alzheimer’s disease	149	52.5
	Dementia in Parkinson’s disease	6	2.1
	Frontotemporal dementia	33	11.6
	Lewy body dementia	5	1.8
	Vascular dementia	21	7.4
	Mild cognitive disorder	31	10.9
	Alcohol dementia	6	2.1
	Other dementia	2	0.7
Medications		274 (10)	
MMSE			
	Yes	233 (51)	82.0
	Not performed	44	15.5
	Not testable	7	2.5
MMSE score		233	
Accommodation			
	Ordinary housing (1)	269	94.4
	Nursing home, temporary (2)	9	3.2
	Nursing home, permanent – not adapted (2)	6	2.1
	Nursing home, permanent- adapted (2)	1	0.4
Living arrangements		277 (7)	
	With another adult (2)	194	70.0
	Living alone (1)	83	30.0
Children at home		267 (17)	
	Yes (1)	24	9.0
	No (2)	243	91.0
Care setting			
	Primary care centres	23	8.1
	Memory clinics	261	91.9

### Support

Regarding support received before diagnosis, only a small percentage had daycare services (1.5%), services regulated by the LSS (2.2%) or home help services (8.8%).

Table 3 displays support offered post-diagnosis for persons with young-onset dementia. Most were offered information and educational support (90.1% for persons with dementia, 78.9% for family members), while around half were offered contact with a dementia nurse (49.3%), a counsellor (51.4%) or a needs assessor (47.9%). Additionally, a third (28.5%) were offered cognitive aids.

**Table 3:** Pre- and Post- Diagnostic Support for young-onset dementia

	Response options (code)	*N* = 284 (missing)	Valid per cent (%)
Support registered at baseline			
Home help service		274 (10)	
	Yes (1)	24	8.8
	No (2)	250	91.2
Daycare		276 (8)	
	Yes, adapted for dementia	2	0.7
	Yes, adapted for younger persons with dementia	1	0.4
	Yes, not adapted for dementia	1	0.4
	No	272	98.6
Support LSS		267 (17)	
	Yes (1)	6	2.2
	No (2)	261	91.9
Support offered by the registration unit			
Information and education (person)			
	Yes (1)	256	90.1
	No (2)	28	9.9
Information and education (family members)			
	Yes (1)	224	78.9
	No (2)	55	19.0
	No relative	5	1.8
Contact needs assessor			
	Yes (1)	136	47.9
	No (2)	148	52.1
Contact dementia nurse			
	Yes (1)	140	49.3
	No (2)	120	42.3
	No, not available	24	8.5
Contact counsellor			
	Yes (1)	146	51.4
	No (2)	138	48.6
Cognitive aids			
	Yes (1)	81	28.5
	No (2)	203	71.5

### Regression analysis

Six regression models (Table 4) predicted the likelihood of offered support for persons with young-onset dementia based on various personal characteristics. The total number of cases in each model and the degrees of freedom are presented. Chi-square was used to determine the goodness of fit, and the Adjusted R^2^ (Nagelkerke) indicated the amount of variance in the outcome variable explained by the model. Post-diagnostic support included *information and education (person and family member*), *dementia nurse*, *needs accessor*, *counsellor*, *cognitive aids, and care setting*. Logistic regression revealed associations between predictor variables and support types.

*Age*, *sex, and* the presence of *children at home* did not show significant associations with the support variables. Notably, *children at home* was found to be collinear with other predictors (Table 4) and was removed from the regression model predicting *information and education* to the person to ensure the validity of the analysis.

*MMSE scores* significantly predicted the likelihood of being offered contact with a needs assessor, with lower scores correlating with a higher likelihood (*p* < .05). However, MMSE scores did not significantly predict other support types.

Neither *medications* nor *accommodation* exhibited significant associations with the support variables. Furthermore, *accommodation* showed collinearity with the *counsellor* and *cognitive aids* variables and was consequently excluded from these regression analyses.

*Living together* significantly predicted the offer of *information and education* for family members (*p* < .01), implying that persons with young-onset dementia living with another adult were more likely to be offered support for their family members. However, *living together* did not significantly predict any other support variables.

*Home help services* were significantly associated with the offer of a *needs assessor* (*p* < .05), indicating that persons already receiving home help services were more likely to be offered contact with a needs assessor. However, *home help services* did not significantly predict any other forms of support.

*Care setting* significantly predicted *information and education* for both the person and family members (*p* < .01), as well as *counsellor*, suggesting that those diagnosed within memory clinics were more likely to be offered support in the form of information and education for themselves and their family members, as well as offered contact with a counsellor.

Three of the six regression models explained a significant proportion of variance in the support variables. Persons living with someone and diagnosed by memory clinics were more likely to be offered information and educational support for their family members. Additionally, those diagnosed by memory clinics were more likely to have been offered contact with a counsellor. Those with lower MMSE scores and those currently utilising home help services were more likely to be offered contact with a needs assessor.

**Table 4:** Results of logistic regressions predicting support offered to persons with young-onset dementia

Variables	Information and education (person) B (SE)	Information and education (family member) B (SE)	Dementia Nurse B (SE)	Needs assessor B (SE)	Counsellor B (SE)	Cognitive aids B (SE)
*Predictors*						
Age	− 0.010 (0.058)	− 0.003 (0.052)	− 0.036 (0.036)	− 0.058 (0.036)	0.074 (0.039)+	− 0.043 (0.039)
Sex	0.590 (0.558)	− 0.069 (0.399)	0.246 (0.304)	− 0.185 (0.305)	0.597 (0.311)+	0.009 (0.325)
Children at home	---^a^	0.905 (1.113)	− 0.061 (0.609)	− 0.026 (0.664)	1.798 (0.953)^+^	− 0.070 (0.725)
MMSE points	0.099 (0.069)	0.024 (0.045)	− 0.026 (0.034)	0.075 (0.035)*	0.019 (0.035)	0.048 (0.036)
Medications	− 0.167 (0.107)	0.003 (0.061)	− 0.021 (0.048)	− 0.0.34 (0.050)	0.075 (0.053)	0.025 (0.056)
Accommodation	0.390 (1.444)	0.576 (1.088)	-1.394 (1.172)	-1.282 (1.241)	---^a^	---^a^
Living together	0.159 (0.614)	-1.020 (0.413)**	0.066 (0.341)	0.217 (0.340)	− 0.019 (0.38)	0.245 (0.358)
Home help service	− 0.448 (0.972)	− 0.214 (0.608)	0.362 (0.562)	1.466 (0.634)*	− 0.110 (0.597)	− 0.118 (0.623)
Care setting	-2.135 (0.741)**	-1.567 (0.576)**	---^a^	− 0.118 (0.592)	-2.982 (1.070)**	-2.023 (1.058)^+^
*Model statistics*						
N	218	205	189	208	208	208
Chi-square	13.47	23.69**	5.83	23.63**	38.87**	10.62
Degrees of freedom	8	9	8	8	8	8
Adjusted R^2^ (Nagelkerke)	13.8%	17.5%	4.1%	14.4%	22.7%	0.7%

^a^ Predictor was colinear with the outcome, preventing its inclusion in the regression.

+ *p* < .10.

* *p* < .05.

** *p* < .01.

## Discussion

This study aimed to describe formal support offered to persons with young-onset dementia and to identify factors influencing support services offered post-diagnosis. Findings showed that the offer of formal support was generally equal, with few significant disparities. Key results indicated minimal support pre-diagnosis, while high levels of information and educational support were offered to the person with young-onset dementia and their family members post-diagnosis. However, it was noted that there was a lack of offered contact with care professionals and offers of cognitive aids. Demographic characteristics had a limited impact on the post-diagnosis support offered.

### Pre-diagnostic formal support

In our sample, persons with young-onset dementia typically lacked pre-diagnostic support, possibly due to persons with early-stage symptoms not yet requiring services like home help, daycare, or services within LSS. However, previous research shows that delays in diagnosis are common [3, 35], and functional disturbances can occur before diagnosis for persons with young-onset dementia [36], indicating a need for pre-diagnostic support. Accessing such services can be challenging without a formal diagnosis, requiring persons with young-onset dementia and their families to navigate the healthcare system independently without prior knowledge or contacts. In line with previous research, this supports earlier recognition and diagnosis of young-onset dementia to provide support [37]. This study also highlights the importance of investigating whether the low rate of support cases masks an unmet need for formal support services. Thus, further research on pre-diagnostic support is essential to understand the needs of persons with young-onset dementia.

### Post-diagnostic formal support

Information and educational support were offered to 90.1% of persons diagnosed with young-onset dementia and 78.9% of their family members. While the National Board of Health and Welfare [30] recommends educational and support programs for all family members of persons with dementia, the SveDem registry lacks specificity regarding the type and extent of information and education provided to persons and their families. Consequently, it remains unclear whether and to what degree these services are utilised and what they consist of. Existing research suggests that while information is provided, it is often written and primarily aimed at family caregivers [12]. Thus, the support services identified in this study may not be effectively tailored to meet the specific needs of persons with young-onset dementia and their families.

Our findings further revealed that while information and educational support offered to family members is generally high (78.9%), families with children at home are not offered more support than those without children, despite potential increased needs. The National Board of Health and Welfare [30] recommends individually tailored support and education for young family members of persons with young-onset dementia. Coping with a parent with young-onset dementia can be challenging, characterised by feelings of uncertainty and grief [38]. Families may delay involving their children in the early stages as they process the diagnosis and strive to maintain normalcy. Additionally, children at home may not be the primary focus of support, especially if the person with young-onset dementia has a spouse providing care. Research shows that children of persons with young-onset dementia often lack adequate support and feel ignored by healthcare systems, relying on the other parent for information [39]. Our findings indicate that if the person with young-onset dementia lives with another adult, there is an increased likelihood of information and educational support being offered to family members, likely due to increased visibility of their needs during appointments when accompanying the person. However, it is essential not to overlook support for relatives who do not reside with the person with young-onset dementia. These family members may also require support due to the burden and distress associated with caring for someone with young-onset dementia [40–42]. According to Aspö et al. [43], family members not living with a person with young-onset dementia have reported an increasing responsibility and a need for support to understand the situation better and prepare for the future. Hence, formal care for persons with young-onset dementia should adopt a family-centred approach, recognising that this diagnosis affects the entire family, regardless of living arrangements.

### Offered contacts

In the study, approximately half of the sample had been offered services by a care professional, such as dementia nurses and needs assessors. This result can be understood in various ways. Receiving a dementia diagnosis could be a shocking and stressful event [11]. Persons with young-onset dementia may initially need time to adjust and focus on the present before seeking support and care and early planning for future care is not always their priority [44]. Moreover, persons in the early stages of dementia may not perceive the need for support [37]. Consequently, those with young-onset dementia may struggle to perceive their support needs and available support services [45].

When the person with young-onset dementia eventually requires support but has not established any previous contacts with care professionals, they may not know where to turn. Regular follow-ups with care professionals could help persons with young-onset dementia to access support services promptly when needed. To ensure adequate support for persons with young-onset dementia, the professional offering support should do so respectfully, refraining from implying immediate need. Instead, they should clarify that services will be available as needed. Such an approach has been shown to enhance the person’s sense of self-agency and capacity, as seen in relationship-centred care [46].

Early interventions play a crucial role in the care of support for persons with young-onset dementia, facilitating optimal participation in future care planning as the condition progresses [44]. Prioritising early contact with municipal care professionals, such as dementia nurses and needs assessors, is essential for improving support for persons with young-onset dementia. This facilitates tailored care and support, even if formal support is not immediately required. Given these considerations regarding offered contacts with care professionals, further research is needed to develop timely support interventions for formal caregivers in determining when to offer such contacts.

In our study, 51.4% reported being offered contact with a counsellor. Persons with young-onset dementia may require assistance in developing skills to handle the emotional impact of their diagnosis [24, 47], highlighting the importance of formal caregivers to prioritise their well-being. Counselling has been shown to have positive effects on depression, anxiety, and overall quality of life for both persons with dementia and their families [48]. Additionally, psychotherapeutic interventions can help persons with dementia manage negative emotions associated with receiving a diagnosis [49]. Given these benefits, contact with a counsellor should have been more widely offered in our study. In Sweden, counsellors also provide support in navigating various aspects of the situation, such as state insurance funds and employers [50], making it a valuable contact to be offered post-diagnosis. One possible explanation for our findings could be Sweden’s lack of specialised counsellors [51]. This could result in certain forms of support being unavailable, with care professionals unable to offer contact with a counsellor.

### Cognitive aids

Cognitive aids, offered to 28.5% of our sample, are a notable aspect of support highlighted in our study. While research on their effectiveness for managing memory problems in persons with young-onset dementia is scarce, studies on assistive technology are increasing [52]. The National Board of Health and Welfare [30] recommends individually adapted cognitive aids based on proven experience. Memory problems pose a significant challenge for persons with young-onset dementia and their families, and cognitive aids can assist in managing appointments, time, and daily tasks, providing a greater sense of security for the person and relief for family members [53]. Despite these potential benefits, most formal caregivers in our study did not offer this support post-diagnosis. This could be due to memory problems not being identified as a priority in the early stages of dementia. Additionally, persons with young-onset dementia may present atypical symptoms such as behavioural changes [2], complicating the identification of their need for these tools. Moreover, memory aid functions are available on mobile phones today, which may be sufficient in earlier stages of dementia and may be perceived as less stigmatising for persons with young-onset dementia.

### Disparities in support for persons with young-onset dementia

Despite extensive research showing gender disparities in dementia care [54, 55], with women receiving less support than men [56], our study found no evidence of such disparities. Previous research has suggested gender inequality in healthcare, particularly favouring men in primary- and hospital care [57, 58]. However, our study indicates gender equality in support for persons with young-onset dementia. This finding could be attributed to various factors, such as the national guidelines for dementia emphasising gender-equal care [30] and increasing equality in legislation regarding support and care.

However, disparities in support based on care setting were evident in our findings, with memory clinics offering more support than primary care centres, reflecting their expertise in working with young-onset dementia. To address this, primary care should transfer patients suspected of young-onset dementia to memory clinics for further evaluation and diagnosis, which aligns with current practice. Although national guidelines [30] state that younger persons are often referred to memory clinics for extended diagnostic workups, they do not explicitly recommend diagnosing young-onset dementia in memory clinics. However, our findings suggest that younger persons benefit from receiving a diagnosis in a memory clinic. Additionally, a US study highlighted positive caregivers’ experiences with memory clinic support, associated with improved health outcomes [59], underscoring the benefits of memory clinics for the family members of the person with young-onset dementia. We also acknowledge memory clinics’ capacity for extended diagnostic workups. Based on these findings, we emphasise the valuable support provided by memory clinics for persons with young-onset dementia and their families. Conducting a follow-up study to assess whether persons with young-onset dementia continue receiving primary care treatment or will be referred to memory clinics could examine whether these disparities persist over time.

### Limitations

Although SveDem’s primary objective is to enhance the quality of care, it also gathers individual-based data for research [34], similar to other Swedish quality registries [60]. However, interpreting these variables for research purposes can be challenging, as they are primarily intended for internal quality assessments by care units. To address this, we thoroughly reviewed the registry’s operations, including relevant documentation and had discussions with the registry administrator to get an insight into variables included in the study. Nonetheless, the support variables included in SveDem have limitations. For instance, it is unclear if persons received or declined support, as entries are often binary (yes/no). Quality registry data is usually simplified to be user-friendly as healthcare professionals entering the data must be able to do so as a part of their daily routine; thus, having detailed variables would risk reducing the number of registrations entered. Moreover, understanding these variables may be challenging for personnel entering data. To ensure the quality of registrations, SveDem is being monitored continuously to maintain data consistency with medical records [34].

Our study had a small sample size due to significant changes in SveDem variables in April 2021. Small sample sizes limit generalisability and potentially introduce demographic bias. However, using the SveDem registry, which covers 100% of memory clinics [33], improves representation compared to primary data collection. Consequently, using quality registries in research comes with limitations but also important strengths, such as improved ability to collect data about populations typically underrepresented in research [61]. Even so, the lack of geographic information on participating units may lead to a clustering effect and impact representativeness. Furthermore, results may not fully represent persons with young-onset dementia, as units affiliated with SveDem might offer better patient support due to regular feedback from SveDem on their results. Persons with young-onset dementia are typically referred to memory clinics for diagnosis, leading to a higher representation of registrations from these clinics in our sample compared to primary care centres [34]. The 2021 SveDem report [33] also notes considerable regional variations in baseline registrations, including potential geographic disparities. Acknowledging that our data lacks important characteristics that could influence the results is important. Although our research aimed to analyse formal support for persons with young-onset dementia and related factors, factors such as socioeconomic status, educational background, and cultural differences were absent from our data for analysis.

SveDem has been criticised for lacking self-reported outcome measures [34] and reflecting only the perspective of care professionals. While there is a self-reported measure regarding support in the registry for the follow-up module, the baseline module used in our study lacks this variable. Incorporating self-reported outcome measures can enhance registry quality by promoting shared decision-making [62], aligning with the goal of person-centred care in Swedish dementia care [30]. Including self-reported outcomes on support needs would have improved the validity of our study. To fully capture the subjective views of persons with young-onset dementia, future research should also prioritise obtaining their perspectives, potentially through interviews.

## Conclusion

This study revealed that while most persons with young-onset dementia and their families were offered information and educational support, the specifics of this support remain unclear. Additionally, there was a notable lack of other forms of support offered post-diagnosis, suggesting that formal support for this group may be inadequate. Notably, the support services offered post-diagnosis appeared consistent across personal characteristics, indicating equality within the sample. However, significant disparities were observed in support services based on care setting. Furthermore, our study indicated minimal utilisation of formal pre-diagnostic support services, underscoring the need for early diagnosis to access these services.

Identified shortcomings highlight the need for further research with a larger sample size to assess their generalisability to a broader population. A longitudinal approach using SveDem follow-up data could provide valuable insight into the evolving support needs of persons with young-onset dementia over time. Additionally, qualitative studies are essential to uncover any unmet support needs concealed within the lack of formal support, both pre-and post-diagnosis. This study serves as a valuable foundation for further research, offering insights into the formal support available for persons with young-onset dementia and their families in Sweden.

## Data Availability

Regrettably, publicly sharing the data is impossible since it was obtained solely from the SveDem registry for this project. Researchers seeking to access the data must obtain ethical approval to use SveDem (http://www.etikprovningsmyndigheten.se). After receiving approval, they can reach out to the data holder for SveDem (https://www.ucr.uu.se/sv/tjanster/blanketter-och-dokument) to request access to the data.

## Abbreviations

SveDem: The Swedish Registry of Cognitive/Dementia Disorders
SoL: The Social Services Act
HSL: The Health and Medical Care Act
LSS: The Act on Support and Service to Certain Functional Impairments
MMSE: The Mini-Mental State Examination

## References

[1] World Health Organization (2021). Global status report on the public health response to dementia.

[2] Draper B, Withall A (2016). Young onset dementia. Intern Med J.

[3] Loi SM, Goh AMY, Mocellin R, Malpas CB, Parker S, Eratne D (2022). Time to diagnosis in younger-onset dementia and the impact of a specialist diagnostic service. Int Psychogeriatr.

[4] Stamou V, La Fontaine J, Gage H, Jones B, Williams P, O’Malley M (2021). Services for people with young onset dementia: the ‘Angela’ project national UK survey of service use and satisfaction. Int J Geriatr Psychiatry.

[5] Kandiah N, Wang V, Lin X, Nyu MM, Lim L, Ng A (2016). Cost related to Dementia in the Young and the impact of Etiological Subtype on cost. J Alzheimers Dis.

[6] Vieira RT, Caixeta L, Machado S, Silva AC, Nardi AE, Arias-Carrion O (2013). Epidemiology of early-onset dementia: a review of the literature. Clin Pract Epidemiol Ment Health.

[7] Withall A, Draper B, Seeher K, Brodaty H (2014). The prevalence and causes of younger onset dementia in Eastern Sydney, Australia. Int Psychogeriatr.

[8] Hendriks S, Peetoom K, Bakker C, Koopmans R, van der Flier W, Papma J (2023). Global incidence of young-onset dementia: a systematic review and meta-analysis. Alzheimer’s Dement.

[9] Skovdahl K, Palo-Bengtsson L, Anttila S, Höjgård U, Fredriksson m, Jonsson A-K (2017). Yngre personer med demenssjukdom och närstående till Dessa Personer - En kunskapssammanställning.

[10] Hendriks S, Peetoom K, Bakker C, van der Flier WM, Papma JM, Koopmans R (2021). Global prevalence of Young-Onset Dementia: a systematic review and Meta-analysis. JAMA Neurol.

[11] Clemerson G, Walsh S, Isaac C (2014). Towards living well with young onset dementia: an exploration of coping from the perspective of those diagnosed. Dementia.

[12] Aspö M, Visser L, Kivipelto M, Boström A-M, Cronfalk BS (2023). Transitions: experiences of younger persons recently diagnosed with Alzheimer-type dementia. Dementia.

[13] Bannon S, Reichman M, Popok P, Wagner J, Gates M, Uppal S (2020). In it together: a qualitative Meta-synthesis of common and unique psychosocial stressors and adaptive coping strategies of persons with Young-Onset Dementia and their caregivers. Gerontologist.

[14] O’Malley M, Carter J, Stamou V, LaFontaine J, Oyebode J, Parkes J (2021). Receiving a diagnosis of young onset dementia: a scoping review of lived experiences. Aging Ment Health.

[15] Popok PJ, Reichman M, LeFeber L, Grunberg VA, Bannon SM, Vranceanu A-M (2022). One diagnosis, two perspectives: lived experiences of persons with Young-Onset Dementia and their care-partners. Gerontologist.

[16] Birt L, Griffiths R, Charlesworth G, Higgs P, Orrell M, Leung P (2020). Maintaining social connections in dementia: a qualitative synthesis. Qual Health Res.

[17] Cipriani G, Danti S, Picchi L, Nuti A, Fiorino MD (2020). Daily functioning and dementia. Dement Neuropsychol.

[18] Millenaar JK, Bakker C, Koopmans RTCM, Verhey FRJ, Kurz A, de Vugt ME (2016). The care needs and experiences with the use of services of people with young-onset dementia and their caregivers: a systematic review. Int J Geriatr Psychiatry.

[19] Spreadbury JH, Kipps C (2019). Measuring younger onset dementia: what the qualitative literature reveals about the ‘lived experience’ for patients and caregivers. Dementia.

[20] Evans D (2016). An exploration of the impact of younger-onset dementia on employment. Dementia.

[21] Livingston G, Sommerlad A, Orgeta V, Costafreda SG, Huntley J, Ames D (2017). Dementia prevention, intervention, and care. Lancet.

[22] Sansoni J, Duncan C, Grootemaat P, Capell J, Samsa P, Westera A (2016). Younger Onset Dementia: a review of the literature to inform Service Development. Am J Alzheimer’s Disease Other Dementias®.

[23] Stamou V, Oyebode J, La Fontaine J, O’Malley M, Parkes J, Carter J. Good practice in needs-based post-diagnostic support for people with young onset dementia: findings from the Angela Project. Ageing Soc. 2023:1–24. 10.1017/S0144686X22001362 PMC7616546 39417728

[24] Grunberg VA, Bannon SM, Reichman M, Popok PJ, Vranceanu A-M (2021). Psychosocial treatment preferences of persons living with young-onset dementia and their partners. Dementia.

[25] Giebel C, Eastham C, Cannon J, Wilson J, Wilson J, Pearson A (2020). Evaluating a young-onset dementia service from two sides of the coin: staff and service user perspectives. BMC Health Serv Res.

[26] Cations M, Withall A, Horsfall R, Denham N, White F, Trollor J (2017). Why aren’t people with young onset dementia and their supporters using formal services? Results from the INSPIRED study. PLoS ONE.

[27] Hälso-. och sjukvårdslag (SFS 2017:30). Stockholm: Socialdepartementet.

[28] Socialtjänstlag. (SFS:2001:453). Stockholm: Socialdepartementet.

[29] Lag om stöd. Och service till vissa funktionshindrade (SFS: 1993:387). Stockholm: Socialdepartementet.

[30] Socialstyrelsen (2017). Nationella Riktlinjer för vård och omsorg vid demenssjukdom - Stöd för styrning och ledning.

[31] Cations M, Loi SM, Draper B, Swaffer K, Velakoulis D, Goh AMY (2021). A call to action for the improved identification, diagnosis, treatment and care of people with young onset dementia. Aust N Z J Psychiatry.

[32] Grunberg VA, Bannon SM, Popok P, Reichman M, Dickerson BC, Vranceanu A-M (2022). A race against time: couples’ lived diagnostic journeys to young-onset dementia. Aging Ment Health.

[33] Svenska registret för kognitiva sjukdomar/demenssjukdomar (2021). Årsrapport 2021.

[34] Religa D, Fereshtehnejad S-M, Cermakova P, Edlund A-K, Garcia-Ptacek S, Granqvist N (2015). SveDem, the Swedish Dementia Registry – A Tool for improving the quality of Diagnostics, Treatment and Care of Dementia patients in clinical practice. PLoS ONE.

[35] Kvello-Alme M, Bråthen G, White LR, Sando SB (2021). Time to diagnosis in Young Onset Alzheimer’s Disease: a Population-based study from Central Norway. J Alzheimers Dis.

[36] Hendriks S, Peetoom K, Tange H, van Bokhoven MA, van der Flier WM, Bakker C (2022). Pre-diagnostic symptoms of Young-Onset Dementia in the General practice up to five years before diagnosis. J Alzheimers Dis.

[37] Eriksson H, Fereshtehnejad S-M, Falahati F, Farahmand B, Religa D, Eriksdotter M (2014). Differences in routine clinical practice between early and late Onset Alzheimer’s disease: data from the Swedish Dementia Registry (SveDem). J Alzheimers Dis.

[38] Sikes P, Hall M (2018). The impact of parental young onset dementia on children and young people’s educational careers. Br Edu Res J.

[39] Groennestad H, Malmedal W (2022). Having a parent with early-onset dementia: a qualitative study of Young Adult Children. Nurs Res Pract.

[40] Johannessen A, Helvik AS, Engedal K, Thorsen K (2017). Experiences and needs of spouses of persons with young-onset frontotemporal lobe dementia during the progression of the disease. Scand J Caring Sci.

[41] Lim L, Zhang A, Lim L, Choong T-M, Silva E, Ng A (2018). High caregiver Burden in Young Onset Dementia: what factors need attention?. J Alzheimers Dis.

[42] Kang M, Farrand S, Walterfang M, Velakoulis D, Loi SM, Evans A. Carer burden and psychological distress in young-onset dementia: an Australian perspective. Int J Geriatr Psychiatry. 2022;37(7). 10.1002/gps.5765 PMC9328388 35708197

[43] Aspö M, Visser LNC, Kivipelto M, Boström AM, Seiger Cronfalk B (2023). Family Members’ experiences of Young-Onset Dementia: becoming responsible yet feeling powerless. J Multidiscip Healthc.

[44] Van Rickstal R, De Vleminck A, Aldridge MD, Morrison SR, Koopmans RT, van der Steen JT (2019). Limited engagement in, yet clear preferences for advance care planning in young-onset dementia: an exploratory interview-study with family caregivers. Palliat Med.

[45] Bamford C, Wheatley A, Brunskill G, Booi L, Allan L, Banerjee S (2021). Key components of post-diagnostic support for people with dementia and their carers: a qualitative study. PLoS ONE.

[46] Nolan MR, Davies S, Brown J, Keady J, Nolan J (2004). Beyond ‘person-centred’ care: a new vision for gerontological nursing. J Clin Nurs.

[47] Rabanal LI, Chatwin J, Walker A, Maria OS, Williamson T. Understanding the needs and experiences of people with young onset dementia: a qualitative study. BMJ Open. 2018;8(10). 10.1136/bmjopen-2017-021166 PMC6196838 30344167

[48] Shoesmith E, Griffiths AW, Sass C, Charura D (2022). Effectiveness of counselling and psychotherapeutic interventions for people with dementia and their families: a systematic review. Ageing Soc.

[49] Sukhawathanakul P, Crizzle A, Tuokko H, Naglie G, Rapoport MJ (2021). Psychotherapeutic interventions for dementia: a systematic review. Can Geriatr J.

[50] Socialstyrelsen [Internet]. Legitimation för kuratorer inom hälso- och sjukvård Stockholm: Socialstyrelsen; 2014 [cited 2023 Dec 09]. https://www.socialstyrelsen.se/globalassets/sharepoint-dokument/artikelkatalog/ovrigt/2014-4-21.pdf.

[51] Socialstyrelsen [Internet]. Bedömning av tillgång och efterfrågan på legitimerad personal i hälso- och sjukvård samt tandvård. Nationella planeringsstödet 2022. Stockholm: Socialstyrelsen; 2023 [cited 2023 Okt 15]. https://www.socialstyrelsen.se/globalassets/sharepoint-dokument/artikelkatalog/ovrigt/2022-2-7759.pdf.

[52] Van der Roest HG, Wenborn J, Pastink C, es RM, Orrell M (2017). Assistive technology for memory support in dementia. Cochrane Database Syst Reviews.

[53] Arntzen C, Holthe T, Jentoft R (2016). Tracing the successful incorporation of assistive technology into everyday life for younger people with dementia and family carers. Dement (London England).

[54] Bartlett R, Gjernes T, Lotherington AT, Obstefelder A (2018). Gender, citizenship and dementia care: a scoping review of studies to inform policy and future research. Health Soc Care Commun.

[55] Alzheimer’s Association. 2014 Alzheimer’s disease facts and figures. Alzheimer’s & Dementia. 2014;10(2):e47-e92. 10.1016/j.jalz.2014.02.001 24818261

[56] Gibbons C, Creese J, Tran M, Brazil K, Chambers L, Weaver B (2014). The psychological and Health consequences of Caring for a spouse with dementia: a critical comparison of husbands and wives. J Women Aging.

[57] Watson J, Green MA, Giebel C, Darlington-Pollock F, Akpan A (2023). Social and spatial inequalities in healthcare use among people living with dementia in England (2002–2016). Aging Ment Health.

[58] Cooper C, Petersen I, Pham TM, Manthorpe J, Raine R, Lodwick R (2017). Inequalities in receipt of mental and physical health care in people with dementia in the U.K.: role of socioeconomic status, gender and ethnicity. Alzheimer’s Dement.

[59] Liu T-L, Yates TD, Taylor YJ, Rossman W, Mangieri D, Black S (2019). Patient and caregiver outcomes and experiences with Team-based memory care: a mixed methods study. J Appl Gerontol.

[60] Emilsson L, Lindahl B, Köster M, Lambe M, Ludvigsson JF (2015). Review of 103 Swedish Healthcare Quality Registries. J Intern Med.

[61] Johansson L, Finkel D, Lannering C, Aslan AKD, Andersson-Gäre B, Hallgren J (2021). Using aggregated data from Swedish national quality registries as tools to describe health conditions of older adults with complex needs. Aging Clin Exp Res.

[62] Nilsson E, Orwelius L, Kristenson M (2016). Patient-reported outcomes in the Swedish National Quality registers. J Intern Med.

